# Effect of Vertebral Morphology on Radiographic and Symptomatic Lumbar Spinal Stenosis in Patients Undergoing Microendoscopic Decompression Surgery: A Retrospective Cohort Study

**DOI:** 10.7759/cureus.79586

**Published:** 2025-02-24

**Authors:** Kusushi Murai, Shizumasa Murata, Hiroki Iwahashi, Yoshimasa Mera, Toshiya Shitahodo, Shingo Inoue, Kota Kawamura, Aozora Kadono, Yoji Kitano, Hiroshi Yamada

**Affiliations:** 1 Department of Orthopedic Surgery, Wakayama Medical University, Wakayama, JPN; 2 Department of Orthopedic Surgery, Shingu Municipal Medical Center, Shingu, JPN

**Keywords:** lumbar spinal stenosis, magnetic resonance imaging, microendoscopic decompression surgery, radiographic stenosis, symptomatic stenosis, vertebral morphology

## Abstract

Purpose

Lumbar spinal stenosis (LSS) is a degenerative condition characterized by spinal canal narrowing, often leading to nerve compression and significantly impairing quality of life, particularly in older adults. Magnetic resonance imaging (MRI) is the gold standard for diagnosing LSS; however, radiographic evidence of stenosis often does not align with clinical symptoms, complicating management. We explored the relationship between vertebral morphology and both radiographic and symptomatic stenosis, hypothesizing that specific vertebral shapes, particularly oval (O) morphology, increase the risk of symptomatic stenosis.

Methods

This retrospective cohort study included patients who underwent microendoscopic decompression surgery between 2010 and 2022. Vertebral morphology was classified as bean (B), plane (P), or oval (O) based on MRI assessments of the posterior vertebral wall. Radiographic stenosis was identified based on MRI findings using the Schizas classification, whereas symptomatic stenosis required a correlation with clinical symptoms, such as neurogenic claudication or radicular pain confirmed through physical examination and functional diagnostics. Levels with radiographic stenosis that lacked clinical correlation were categorized as asymptomatic stenosis. The primary outcome was the prevalence of radiographic and symptomatic stenoses across different vertebral morphology groups. Secondary outcomes included the influence of patient demographics and morphology on stenosis at various lumbar levels. Statistical analyses included Chi-square tests and multivariate logistic regression, with p-values <0.05 considered significant.

Results

A total of 234 patients (105 females and 129 males; mean age: 72.2 years) were included. Radiographic stenosis prevalence increased progressively from L1/2 to L4/5, with the highest rate at L4/5 (167/234, 71.4%). Among these cases, symptomatic stenosis was observed in 86.2% (144/167), with O morphology showing the strongest association (79/86, 91.2%), followed by P morphology (65/81, 80.2%). By contrast, B morphology had the lowest prevalence of radiographic stenosis (10/103, 9.7%) and the weakest correlation with symptomatic outcomes (2/38, 5.3%). Overall, 265/337 (78.6%) of radiographic stenosis cases were symptomatic, whereas 72/337 (21.4%) remained asymptomatic despite MRI findings, highlighting the gap between imaging findings and clinical symptoms. Multivariate analysis confirmed that O morphology was significantly associated with symptomatic stenosis (odds ratio: 3.45; 95% CI: 2.10-5.67; p<0.001), underscoring the influence of vertebral morphology on symptomatic presentation.

Conclusions

Vertebral morphology was observed to influence the prevalence and severity of both radiographic and symptomatic stenosis. The O morphology demonstrated a higher prevalence of symptomatic stenosis, particularly at the L4/5 level, whereas the B morphology was associated with the lowest prevalence. These findings suggest that incorporating vertebral morphology into diagnostic evaluations and treatment planning for patients with LSS may enhance alignment between imaging findings and clinical presentations, facilitating more accurate prognostic assessments and tailored strategies.

## Introduction

Lumbar spinal stenosis (LSS) is a prevalent degenerative condition characterized by the narrowing of the spinal canal, which can lead to compression of the spinal cord and nerve roots, resulting in significant neurological dysfunction. This condition markedly impairs the quality of life in the aging population, with epidemiological studies reporting a prevalence of LSS in 12-47% of adults over 60 years, highlighting its substantial impact on public health [1]. Magnetic resonance imaging (MRI) remains the gold standard for diagnosing LSS owing to its high-resolution capability, providing detailed visualization of the spinal canal and neural structures [2]. However, interpreting MRI findings is challenging, as radiographic evidence of stenosis often fails to correlate with the patient’s clinical symptoms [3]. Previous studies have highlighted the potential role of paraspinal muscles as an alternative diagnostic tool in such cases. For instance, paraspinal muscle denervation has been strongly correlated with balance impairments in symptomatic patients [4], while paraspinal electromyography findings have demonstrated a better correlation with clinical symptoms than MRI in asymptomatic individuals [5].

The pathophysiology of LSS involves compression of the dural sac and nerve roots, primarily caused by bulging intervertebral discs and hypertrophied ligamentum flavum from degenerative changes. Interestingly, the shape of the intervertebral disc often resembles the shape of the lower endplate of the superior vertebral body until degeneration occurs [6,7]. For example, the morphology of the L4/5 disc typically mirrors the shape of the inferior endplate of the L4 vertebra. As degeneration progresses, disc bulging into the spinal canal leads to nerve compression. We postulate that the initial disc shape may influence the likelihood and pattern of posterior bulging, contributing to differences in the development of symptomatic stenosis. Specifically, we hypothesize that oval-shaped discs may be more prone to posterior bulging and nerve compression than bean-shaped discs, which may exhibit a lower propensity for posterior protrusion despite degeneration.

Previous studies, such as the Wakayama Spine Study, have illustrated discrepancies between radiographic and symptomatic stenosis in LSS cases [8]. This large, community-based study revealed that many asymptomatic individuals had MRI evidence of lumbar stenosis, suggesting a potential overestimation of clinically significant stenosis when relying solely on imaging [8]. Despite the widespread use of the Schizas classification for grading spinal stenosis severity on MRI [9], the association between these radiographic classifications and clinical symptoms remains unclear [10].

A critical gap remains in understanding how vertebral morphology affects the relationship between radiographic and symptomatic stenosis. While previous studies have emphasized the role of paraspinal muscles and their correlation with clinical symptoms [4,5], our study adopts a complementary approach by focusing on vertebral morphology as a potential risk factor for this discrepancy. Although the Wakayama Spine Study provided foundational insights, it did not address how variations in the posterior vertebral wall shape might impact symptomatology [8]. Specifically, data on the clinical relevance of different posterior vertebral wall shapes, categorized as bean (B), plane (P), and oval (O), remain limited [11]. We hypothesize that the morphology of the posterior vertebral wall at the inferior endplate level of the upper vertebra could explain the observed gap between imaging-based and symptomatic stenosis. Moreover, we suggest that bean-shaped walls may provide a protective effect, delaying or reducing nerve compression compared with oval-shaped configurations.

Therefore, this study (1) elucidates the relationship between vertebral morphology and the occurrence of radiographic and symptomatic stenosis and (2) examines whether specific vertebral morphologies are associated with a higher or lower risk of developing symptomatic stenosis. We propose that vertebral shape is a crucial factor influencing the clinical presentation of LSS and that comprehensively evaluating vertebral morphology may enhance the diagnostic and therapeutic approach to this condition, ultimately bridging the gap between MRI findings and clinical symptoms.

## Materials and methods

Ethical considerations

All patient procedures performed in this study were in accordance with the ethical standards of the Research Ethics Committee of Shingu Municipal Medical Center (approval number: 105-2) and with the 1964 Declaration of Helsinki and its later amendments or comparable ethical standards.

Study design and population

This retrospective cohort study was conducted at a key medical center between 2010 and 2022, focusing on patients who underwent microendoscopic decompression surgery for LSS. Inclusion criteria were patients aged ≥18 years who had this surgery at our institution during the study period and had complete medical records, including preoperative and postoperative MRI data. Patients with lumbar disc herniation and foraminal stenosis, which differ pathophysiologically from central LSS, were excluded. These conditions were identified using MRI and clinical criteria. Lumbar disc herniation was excluded based on MRI findings demonstrating focal disc protrusion or extrusion causing nerve root compression, with or without migration, rather than generalized spinal canal narrowing. Foraminal stenosis was ruled out using T2-weighted sagittal and oblique MRI views, where nerve root compression within the foraminal or extraforaminal regions was identified. In addition, patients presenting with clinical symptoms primarily indicative of disc herniation (e.g., acute radicular pain and positive straight leg raise test) or foraminal stenosis were excluded from the study. Additionally, individuals with prior lumbar spine surgery, incomplete medical or imaging records, or substantial comorbidities (e.g., severe neurological disorders or systemic diseases that could interfere with the assessment of LSS symptoms) were excluded.

Data collection

**Figure 1:**
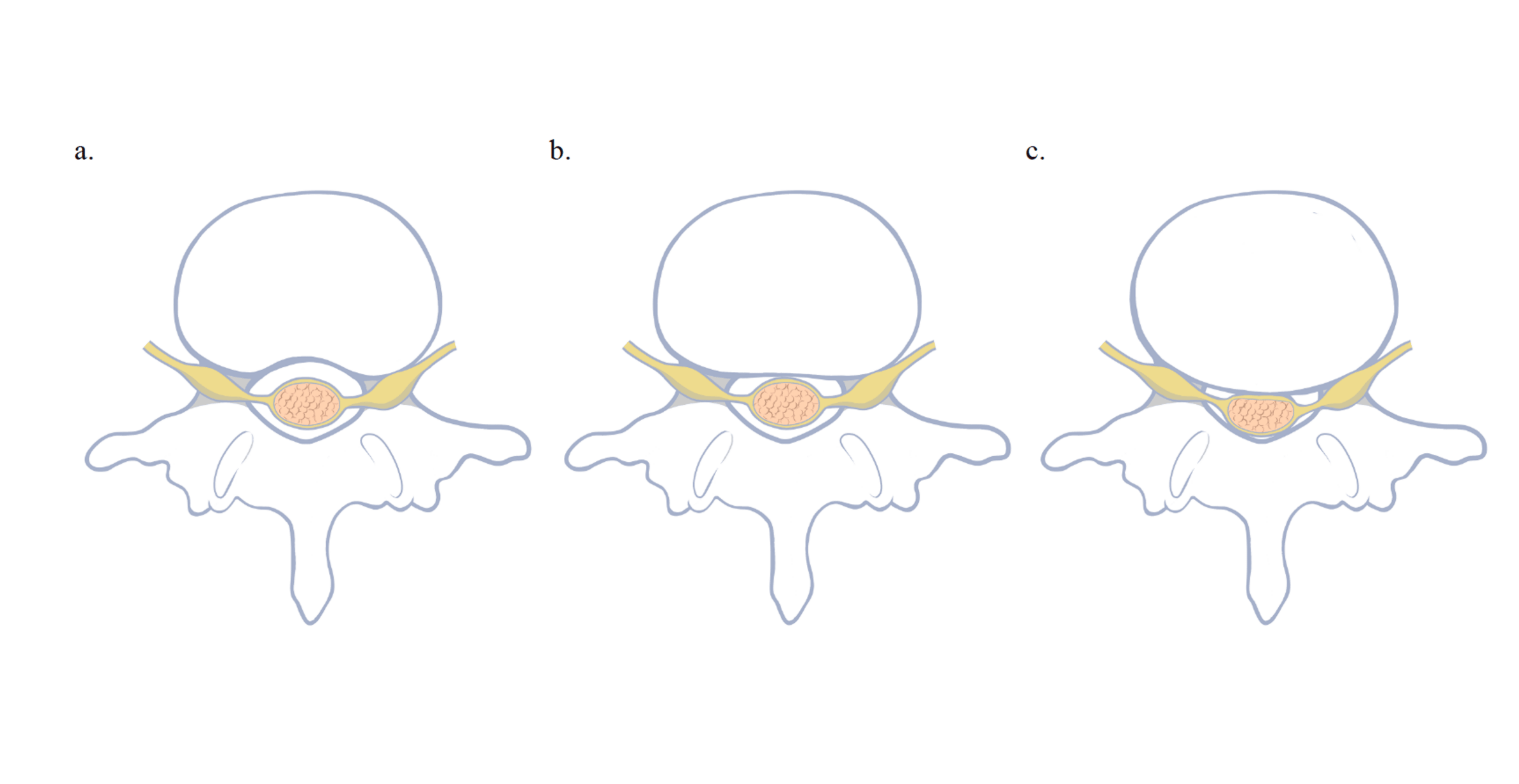
Bean (B), plane (P), and oval (O) morphologies a. Bean type: representing a concave morphology with minimal posterior protrusion. b. Plane type: characterized by a flat posterior vertebral wall. c. Oval type: depicting a rounded posterior vertebral wall with a higher likelihood of posterior protrusion. Image Credit: This figure was created by the authors.

We collected data from the hospital's electronic medical records and imaging databases, including demographic information (age and sex), clinical history, surgical details, and MRI findings. Vertebral morphology and the severity of spinal canal stenosis were assessed using MRI. Specifically, the shape of the posterior vertebral wall at the lower endplate level of the upper lumbar vertebrae was categorized into three types: bean (B), plane (P), and oval (O), as shown in Figure 1.

This classification is based on previous studies showing that vertebral morphology influences disc bulging and subsequent nerve compression [6,7]. No atypical vertebral shapes were observed in this study. All vertebrae were categorized into one of the three predefined morphology types: bean (B), plane (P), or oval (O), ensuring consistency in the classification process.

To ensure assessment reliability, two independent observers evaluated the vertebral morphology and stenosis severity. Intra- and interrater reliability were calculated using Cohen’s kappa (κ) statistics, which demonstrated substantial agreement for both measures. The intrarater reliability for vertebral morphology was κ=0.86 (95% CI: 0.81-0.91) and for stenosis, severity was κ=0.89 (95% CI: 0.84-0.93). The interrater reliability for vertebral morphology was κ=0.83 (95% CI: 0.78-0.88) and for stenosis, severity was κ=0.85 (95% CI: 0.80-0.90), confirming high consistency between observers.

Definitions

Radiographic stenosis was evaluated using MRI and defined based on the Schizas classification, a widely accepted qualitative grading system for LSS severity. It categorizes stenosis into four grades based on the morphology of the dural sac: Grade A: CSF is clearly visible within the dural sac, with rootlets organized in a horseshoe shape. Grade B: Some rootlets are aggregated, but there is still visible CSF within the dural sac. Grade C: No CSF is visible, with all rootlets tightly packed together. Grade D: Severe stenosis, where the dural sac appears homogeneous, with no discernible rootlets or CSF space.

In this study, we defined radiographic stenosis as cases meeting the Schizas Grade C or D criteria to ensure that only moderate-to-severe stenosis cases were included.

Symptomatic stenosis was defined as radiographic stenosis that correlated with clinical symptoms, such as neurogenic claudication or radicular pain, confirmed through physical examination and functional diagnostics, which included nerve conduction studies and selective nerve root blocks. Diagnosing symptomatic stenosis required radiographic evidence of stenosis and positive clinical findings suggesting nerve root compression or irritation.

Asymptomatic stenosis was defined as radiographic stenosis identified on MRI without a consistent correlation with clinical symptoms, such as neurogenic claudication or radicular pain. These cases were characterized by discrepancies between imaging findings and clinical evaluations, where the suspected lesion was not confirmed at the symptomatic level through physical examination, selective nerve root blocks, or electrophysiological testing. Consequently, these levels were deemed unsuitable for surgical intervention.

Each lumbar level (L1/2 to L4/5) was independently assessed for stenosis and categorized into no stenosis, radiographic stenosis, or symptomatic stenosis. Patients were not classified into a single category but had multiple levels assessed separately. All patients included in the study had at least one symptomatic stenotic level that justified surgical intervention.

Outcome measures

The primary outcome measure was the relationship between vertebral morphology and the prevalence of radiographic and symptomatic stenoses in patients undergoing microendoscopic decompression for LSS. We assessed the prevalence of radiographic stenosis in different vertebral morphology groups (B, P, and O), the correlation between radiographic stenosis and symptomatic presentation within each morphology group, and the proportion of cases with radiographic stenosis that did not result in symptoms, focusing on differences across vertebral morphologies.

Secondary outcomes included the association between patient demographics (age, sex, and BMI) and the risk of symptomatic stenosis, the distribution of vertebral morphology types concerning the severity of radiographic stenosis, and the relationship between radiographic and symptomatic stenosis across lumbar levels (L1/2, L2/3, L3/4, and L4/5), considering vertebral morphology's influence.

Statistical analysis

Descriptive statistics were used to summarize demographic and baseline characteristics. Continuous variables, such as age and BMI, are presented as means and standard deviations, whereas categorical variables, such as sex and vertebral morphology, are presented as frequencies and percentages. To assess the association between categorical variables, Chi-square tests were conducted, specifically to evaluate whether the prevalence of radiographic and symptomatic stenosis differed significantly among the B, P, and O vertebral morphology groups.

Additionally, statistical comparisons were made separately for each lumbar level (L1/2, L2/3, L3/4, and L4/5) to determine variations in stenosis prevalence and morphology distribution across these levels. Fisher's exact test was used for small sample sizes to ensure the accuracy of p-values.

Multivariate logistic regression analysis was performed to adjust for potential confounders such as age, sex, and BMI when assessing the association between vertebral morphology and the risk of symptomatic stenosis. Odds ratios (ORs) with 95% CIs were calculated to quantify the strength of these associations. The statistical significance threshold was set at p<0.05, and all analyses were conducted using SPSS software (version 27.0; IBM Corp., Armonk, NY).

Where applicable, 95% CI was reported to provide precision for prevalence estimates and support the observed associations' reliability.

## Results

Patient demographics and characteristics

A total of 265 patients (124 females and 141 males) initially met the inclusion criteria. After excluding 21 patients with concomitant lumbar disc herniation or foraminal stenosis, six with a history of prior lumbar surgery, and four with insufficient MRI imaging (due to cardiac pacemaker placement), 234 patients (105 females and 129 males) were included in the final analysis. The mean age was 72.2 years (standard deviation: 8.3 years), and the average BMI was 23.2±3.7 kg/m². A slightly higher proportion of male patients (129, 55.1%) than female patients (105, 44.9%) was observed, as presented in Table 1.

**Table 1:** Patient demographics and characteristics Values are presented as means with standard deviations and ranges. BMI, body mass index

Characteristic	Value
Total patients	234
Female patients	105 (44.9%)
Male patients	129 (55.1%)
Mean age (years)	72.2±8.3 (range: 42-89)
Mean BMI (kg/m²)	23.2±3.7 (range: 22-30)

**Figure 2:**
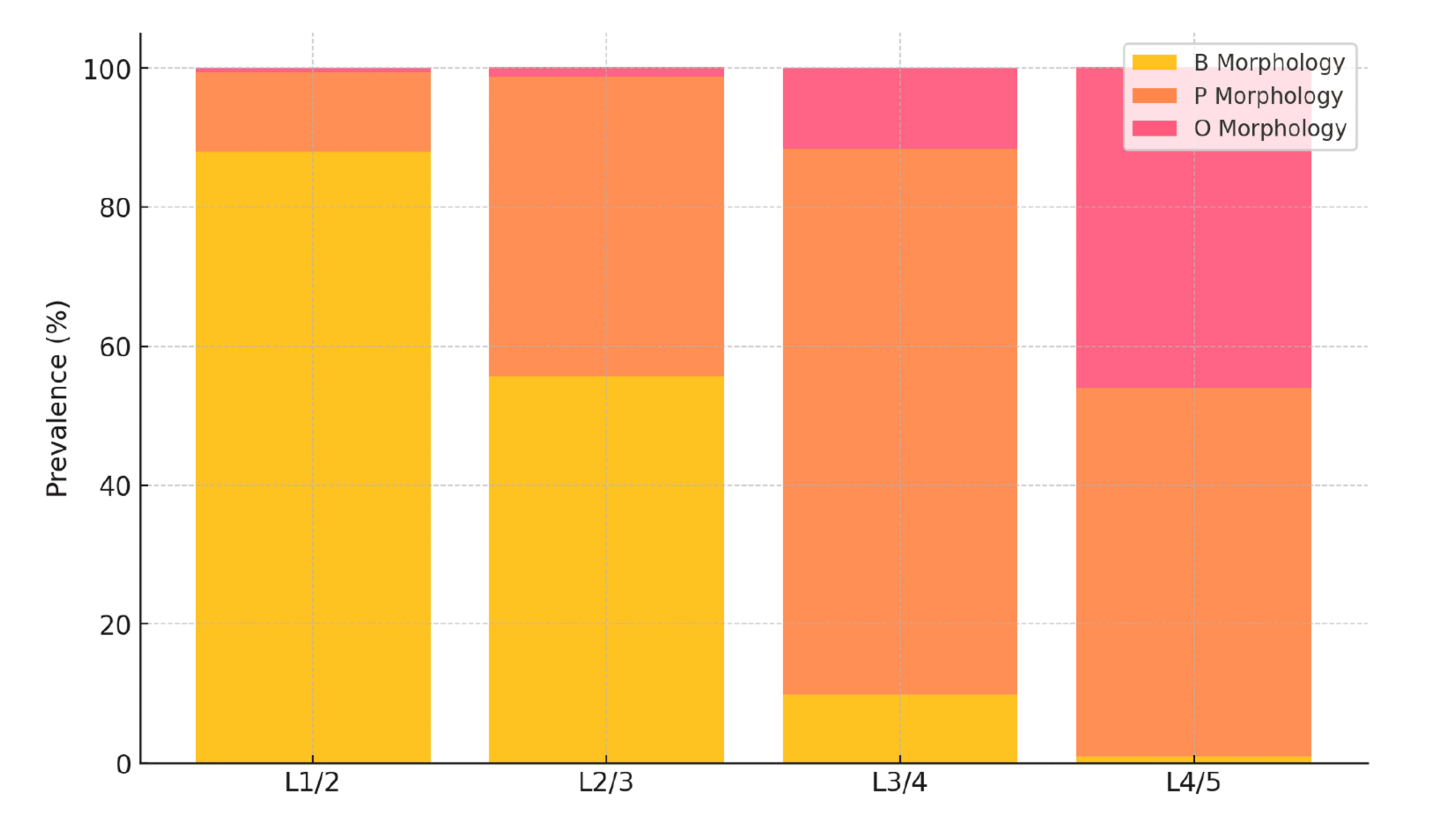
Prevalence of radiographic stenosis and vertebral morphology distribution description (n=234) The stacked bar graph shows the prevalence of radiographic stenosis at each lumbar level (L1/2, L2/3, L3/4, and L4/5), with the distribution of vertebral morphology categorized as B, P, and O types. The B morphology (yellow bars) is predominant at higher lumbar levels, whereas P and O morphologies (orange and pink bars, respectively) become more prevalent at lower lumbar levels. The graph highlights the progressive increase in stenosis prevalence from L1/2 to L4/5.

Radiographic stenosis prevalence varied across different lumbar levels (Figure 2 and Table 2). At the L1/2 level, 4.3% (10/234) of patients had radiographic stenosis, predominantly associated with the B morphology (8/206, 3.9%), with fewer cases in the P morphology (2/28, 7.1%), and none in the O morphology group (0%). At the L2/3 level, 17.5% (41/234) of patients exhibited radiographic stenosis, with a distribution of B morphology (23/130, 17.7%), P morphology (17/101, 16.9%), and O morphology (1/3, 33.3%). The L3/4 level showed an increase, with 50.9% (119/234) presenting with stenosis, primarily in the P morphology group (96/184, 52.2%), followed by O morphology (20/35, 57.1%), and B morphology (3/15, 20.0%). The highest prevalence was observed at the L4/5 level, where 71.4% (167/234) had stenosis, distributed among P morphology (81/124, 65.3%) and O morphology (86/108, 79.6%), with no cases in the B morphology group (0/2, 0%).

**Table 2:** Radiographic stenosis prevalence varied across different lumbar levels

Lumbar level	Total patients with stenosis	B morphology	P morphology	O morphology
L1/2	10/234 (4.3%)	8/206 (3.9%)	2/28 (7.1%)	0 (0%)
L2/3	41/234 (17.5%)	23/130 (17.7%)	17/101 (16.9%)	1/3 (33.3%)
L3/4	119/234 (50.9%)	3/15 (20.0%)	96/184 (52.2%)	20 /35(57.1%)
L4/5	167/234 (71.4%)	0/2 (0%)	81/124 (65.3%)	86/108 (79.6%)

Relationship between radiographic and symptomatic stenoses

Multivariate logistic regression analysis was used to assess the relationship between vertebral morphology and the risk of symptomatic stenosis while adjusting for potential confounders such as age, sex, and BMI. The analysis demonstrated that O morphology had a significant association with symptomatic stenosis compared with B morphology (OR: 3.45; 95% CI: 2.10-5.67; p<0.001), highlighting a potential observational relationship. P morphology also showed a moderate association with symptomatic stenosis (OR: 1.87; 95% CI: 1.15-3.04; p=0.012). These results indicate that vertebral morphology plays a crucial role in predicting symptomatic stenosis, even after controlling for demographic factors (Table 3).

**Table 3:** Multivariate logistic regression analysis of vertebral morphology and symptomatic stenosis Adjusted for age, sex, and BMI. OR, odds ratio; BMI, body mass index

Vertebral morphology	OR	95% CI	p-value
B morphology	Reference	-	-
P morphology	1.87	1.15-3.04	0.012
O morphology	3.45	2.10-5.67	<0.001

Effect of vertebral morphology on symptomatic outcomes

**Figure 3:**
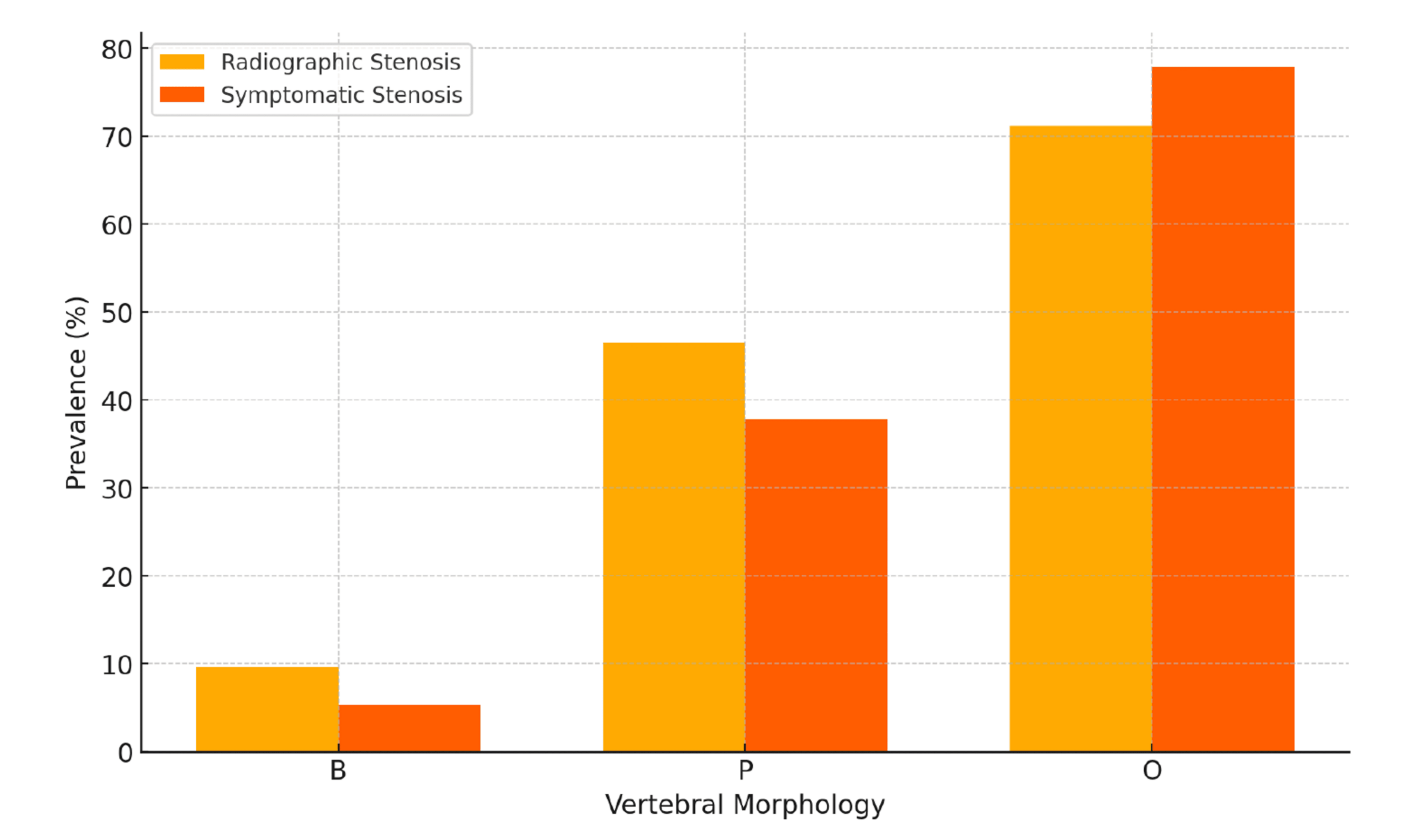
Effect of vertebral morphology on radiographic and symptomatic stenoses description (n=234) The grouped bar graph illustrates the association between vertebral morphology (B, P, and O types) and the prevalence of radiographic and symptomatic stenoses. Radiographic stenosis (yellow bars) and symptomatic stenosis (orange bars) are compared across the three morphologies. The B morphology shows the lowest prevalence of both radiographic and symptomatic stenoses, whereas the O morphology demonstrates the highest risk, indicating a significant influence of vertebral shape on clinical outcomes.

Among patients with B morphology, only 9.6% (34/353) showed radiographic stenosis, and 5.4% (19/353) were symptomatic. By contrast, P morphology had a higher prevalence of radiographic stenosis 44.9% (196/437), with 37.8% (165/437) of patients being symptomatic. The strongest association was observed in patients with O morphology, where 73.3% (107/146) had radiographic stenosis, and 76.9% (113/146) exhibited symptomatic stenosis. The adjusted analysis confirmed that O morphology was the most predictive of symptomatic stenosis, emphasizing the influence of vertebral shape on clinical outcomes (Figure 3).

Prevalence and trends across lumbar levels

**Figure 4:**
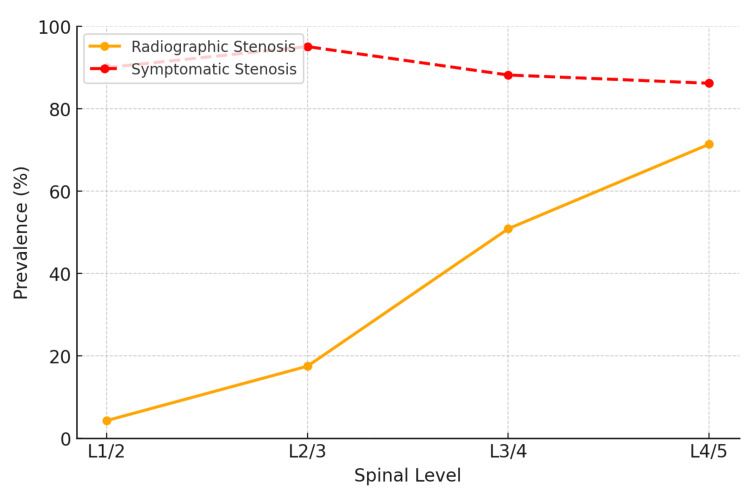
Trends in stenosis prevalence from L1/2 to L4/5 levels description (n=234) The line graph depicts the trends in radiographic (solid line with circles) and symptomatic (dashed line with circles) stenosis prevalence from L1/2 to L4/5 levels. The data show a progressive increase in radiographic stenosis prevalence as the lumbar level descends, with symptomatic stenosis occurring as a subset of radiographic cases. Importantly, not all individuals with radiographic stenosis develop symptoms, highlighting the gap between imaging findings and clinical presentation. This discrepancy becomes more pronounced at the lower lumbar levels, where O morphology is more strongly associated with symptomatic outcomes.

The prevalence of stenosis increased progressively from the L1/2 to the L4/5 level. At the L1/2 level, radiographic stenosis was present in 4.3% (10/234) of patients, and 90.0% (9/10) were symptomatic, predominantly associated with B morphology. At L2/3, the prevalence rose to 17.5% (41/234), with symptomatic stenosis occurring in 95.1% (39/41), and P and O morphologies becoming more prominent. The L3/4 level had 50.9% (119/234) of patients with radiographic stenosis and 88.2% (105/119) with symptomatic stenosis, with O morphology showing a stronger correlation. The highest rates were seen at L4/5, where 71.4% (167/234) of patients had radiographic stenosis and 86.2% (144/167) of these cases were symptomatic, primarily linked to O morphology (79/86, 91.2%), followed by P morphology (65/81, 80.2%). These patterns underscore the increasing influence of vertebral morphology on stenosis prevalence and symptomatology at lower lumbar levels (Figure 4).

## Discussion

This study found a strong correlation between vertebral morphologies and both radiographic and symptomatic stenosis. O morphology was associated with the highest prevalence of stenosis, particularly at the L4/5 level. Conversely, B morphology showed the lowest incidence of stenosis, indicating a protective effect. Notably, 21.4% of patients with radiographic stenosis were asymptomatic, highlighting a disparity between imaging findings and clinical symptoms. This reinforces the need for comprehensive clinical evaluation in patients with LSS.

This study is among the first to systematically investigate the impact of vertebral morphology on both radiographic and symptomatic stenosis across multiple lumbar levels. Identifying the O morphology as a significant predictor of symptomatic stenosis, particularly at lower lumbar levels, contributes to a better understanding of LSS pathophysiology. The rigorous inclusion criteria, large sample size, and multivariate analysis enhance the robustness and generalizability of the findings. Moreover, the distribution of symptomatic stenosis supports the hypothesis that B and P morphologies may provide greater physical and spatial buffering capacity against disc degeneration and posterior bulging, thereby delaying nerve compression compared with O morphology. This interpretation offers a robust anatomical basis for understanding the variability in stenosis progression among different vertebral shapes. However, as the present study employed a cross-sectional design, longitudinal validation of these hypotheses is an important avenue for future research. Prospective studies exploring the dynamic interplay between vertebral morphology, disc degeneration, and stenosis progression are needed to validate these findings and further elucidate their clinical implications.

Our findings align with previous studies, such as the Wakayama Spine Study, which emphasized the complex relationship between radiographic findings and clinical symptoms of LSS. For instance, radiographic evidence often overestimates the prevalence of symptomatic LSS, suggesting that relying on imaging alone may be insufficient for accurate diagnosis. By incorporating vertebral morphology into our analysis, we offer an anatomical explanation for this discrepancy, which is associated with a higher prevalence of symptomatic stenosis, particularly at the L4/5 level. However, this association should be interpreted as observational rather than causal, highlighting the structural characteristics of the O morphology as a potential contributing factor. These findings support the hypothesis that vertebral shape significantly influences clinical presentations by increasing the likelihood of nerve compression under multifactorial pathological conditions, such as ligamentum flavum hypertrophy and intervertebral disc bulging. It is also important to note that spinal stenosis is influenced by various other factors, including activity level, body weight, and genetic predisposition, which were not accounted for in the present study. The factors could potentially interact with vertebral morphology to affect stenosis progression and symptomatology. Future studies should aim to integrate these variables into multivariate analyses to provide a more comprehensive understanding of the multifactorial nature of spinal stenosis and its clinical implications.

Previous research, including studies by Lee et al., has suggested that specific vertebral shapes may predispose individuals to more severe stenosis by altering spinal canal dimensions [12]. Our findings extend this understanding by demonstrating that vertebral morphology is a critical risk factor for symptomatic stenosis, supporting the assertion that anatomical variations can significantly impact clinical outcomes. The O morphology, characterized by a rounded posterior vertebral wall, was associated with an increased prevalence of symptomatic stenosis. This association may stem from its structural characteristics, which potentially affect spinal canal dimensions and load distribution. In particular, the reduced buffering capacity of the O morphology against posterior disc bulging could contribute to neural compression, particularly under degenerative conditions such as ligamentum flavum hypertrophy. However, such interpretations remain speculative and require further biomechanical and longitudinal studies to validate. Additionally, the rounded shape of the posterior vertebral wall in the O morphology may elevate local mechanical stress on neural elements, increasing the risk of symptomatic presentations. Future research employing biomechanical modeling and advanced imaging techniques is necessary to validate these hypotheses and better elucidate the structural and functional contributions of vertebral morphology to stenosis progression. While Schizas et al. focused on dural sac morphology for stenosis classification, our study offers a complementary perspective by emphasizing the clinical relevance of vertebral wall shapes, thereby providing a more nuanced understanding of LSS pathophysiology [10].

Biomechanical considerations further highlight the importance of vertebral morphology. Studies, such as those by Koch et al., have shown how sagittal spinal alignment and vertebral shape affect load distribution, potentially exacerbating stenosis [13-16]. Our findings support this concept, particularly for the O morphology, which may lead to altered load dynamics and increased nerve root compression, explaining the higher prevalence and severity of symptoms observed in patients with this morphology. Genevay and Atlas have also emphasized the variability in LSS presentations, highlighting the need for combining imaging with a thorough clinical assessment for accurate diagnosis [9]. Our results affirm this approach, suggesting that incorporating vertebral morphology into the diagnostic process can improve the alignment between radiographic findings and patient symptoms.

Clinically, our findings have several important implications. Identifying high-risk morphological patterns, such as the O shape, may improve prognostic accuracy and inform surgical planning, particularly in cases where imaging findings and clinical symptoms are incongruent. Specifically, the results provide an opportunity to re-evaluate whether imaging-detected stenosis corresponds to the symptomatic lesion. This approach could prevent the unnecessary expansion of surgical indications and enable clinicians to focus on less invasive surgical techniques targeting the actually responsible lesion. Furthermore, the results could support the development of predictive tools to identify individuals at higher risk of symptomatic stenosis based on vertebral morphology. Once validated through longitudinal studies, such tools could facilitate early screening and guide preventive interventions.

The present study has some limitations. Its retrospective design precludes establishing causal relationships between vertebral morphology and LSS symptoms. Additionally, the single-center nature of the study and its focus on surgical cases may limit the external validity of the results. The exclusion of patients with milder or conservatively managed LSS introduces potential selection bias, limiting generalizability to a broader population. The cohort predominantly consisted of older adults undergoing surgical intervention, which may not fully represent individuals with asymptomatic or less severe disease. Additionally, the present study did not include asymptomatic individuals, whose vertebral morphology could provide critical insights into the natural progression from radiographic findings to symptomatic stenosis. Therefore, future studies should incorporate asymptomatic populations to establish a baseline for vertebral morphology and better elucidate its role in symptom development. Furthermore, a priori power analysis to determine the sample size was not conducted, as the sample was based on the retrospective availability of patients meeting the inclusion criteria. This may reduce the statistical power to detect smaller effect sizes. Furthermore, the axial MRI images used to evaluate vertebral morphology did not consistently include paraspinal muscles, which play a crucial role in LSS. Future studies should ensure the inclusion of paraspinal muscle assessments alongside vertebral morphology to eliminate confounding factors and enhance the accuracy of findings. Another key limitation is the lack of analysis of the Subcutaneous Fat Index (SFI), which could provide additional insights into spinal degeneration. The MRI data used in the present study did not consistently capture the full subcutaneous fat layer, making it infeasible to include SFI in the current analysis. Future studies should ensure comprehensive MRI protocols to facilitate the incorporation of SFI and improve predictive accuracy. Additionally, variability in MRI quality, including differences in slice intervals and resolution, could have influenced the accuracy of vertebral morphology classification and stenosis grading. Standardized imaging protocols across institutions are essential for enhancing the reproducibility of findings in future studies. The establishment of consistent imaging parameters will mitigate discrepancies and improve the reliability of results in multicenter research. The study also did not differentiate between congenital and acquired stenosis. While high-risk congenital conditions, such as achondroplasia, were not present in the cohort, this omission limits the understanding of how congenital factors interact with vertebral morphology to influence clinical outcomes. Finally, symptomatic stenosis was defined on the basis of clinical and imaging findings without incorporating detailed patient-reported outcomes or objective measures of symptom severity. The limitation underscores the need for prospective studies that include patient-reported outcome measures and objective functional assessments. Future prospective, multicenter, and longitudinal studies are essential to validate the findings, address the limitations, and explore the temporal relationships among vertebral morphology, stenosis progression, and symptom onset. Such studies would facilitate a more comprehensive understanding of the multifactorial nature of LSS and its clinical implications.

## Conclusions

Overall, this study highlights the significant role of vertebral morphology, particularly the O shape, in the development of symptomatic LSS. Our findings suggest that specific vertebral shapes predispose patients to more severe clinical symptoms, which has important implications for the diagnosis and management of LSS. By incorporating vertebral morphology into the diagnostic process, clinicians can improve prognostic assessments and optimize treatment strategies, ultimately enhancing patient outcomes.
